# PlantLTRdb: An interactive database for 195 plant species LTR-retrotransposons

**DOI:** 10.3389/fpls.2023.1134627

**Published:** 2023-03-06

**Authors:** Morad M. Mokhtar, Alsamman M. Alsamman, Achraf El Allali

**Affiliations:** African Genome Center, Mohammed VI Polytechnic University, Benguerir, Morocco

**Keywords:** LTR-retrotransposons, plant genomes, database, insertion age, LTR-RT gene chimeras

## Abstract

LTR-retrotransposons (LTR-RTs) are a large group of transposable elements that replicate through an RNA intermediate and alter genome structure. The activities of LTR-RTs in plant genomes provide helpful information about genome evolution and gene function. LTR-RTs near or within genes can directly alter gene function. This work introduces PlantLTRdb, an intact LTR-RT database for 195 plant species. Using homology- and *de novo* structure-based methods, a total of 150.18 Gbp representing 3,079,469 pseudomolecules/scaffolds were analyzed to identify, characterize, annotate LTR-RTs, estimate insertion ages, detect LTR-RT-gene chimeras, and determine nearby genes. Accordingly, 520,194 intact LTR-RTs were discovered, including 29,462 autonomous and 490,732 nonautonomous LTR-RTs. The autonomous LTR-RTs included 10,286 *Gypsy* and 19,176 *Copia*, while the nonautonomous were divided into 224,906 *Gypsy*, 218,414 *Copia*, 1,768 BARE-2, 3,147 TR-GAG and 4,2497 unknown. Analysis of the identified LTR-RTs located within genes showed that a total of 36,236 LTR-RTs were LTR-RT-gene chimeras and 11,619 LTR-RTs were within pseudo-genes. In addition, 50,026 genes are within 1 kbp of LTR-RTs, and 250,587 had a distance of 1 to 10 kbp from LTR-RTs. PlantLTRdb allows researchers to search, visualize, BLAST and analyze plant LTR-RTs. PlantLTRdb can contribute to the understanding of structural variations, genome organization, functional genomics, and the development of LTR-RT target markers for molecular plant breeding. PlantLTRdb is available at https://bioinformatics.um6p.ma/PlantLTRdb.

## 1 Introduction

Long terminal repeats (LTR) have attracted considerable interest in recent years because of their potential impact on the genome structure of most eukaryotic organisms (Jedlicka et al., 2020). LTR-retrotransposons (LTR-RTs) are a large and diverse group of transposable elements (TE) that replicate *via* an RNA intermediate (Jedlicka et al., 2020). LTR-RTs are divided into autonomous and nonautonomous groups. Wicker et al. (2007) defined LTR-RT as autonomous if it encodes all domains essential for its mobilization without the element being either functional or active. This is in contrast to nonautonomous LTR-RT, which are defined as elements that lack some (or all) of the domains necessary for mobilization. Features of an autonomous LTR-RT include two identical LTRs, a primer binding site (PBS), a polypurine tract (PPT), *GAG* and *Pol* genes (Kumar and Bennetzen, 1999; Gao et al., 2003; Havecker et al., 2004; Eickbush and Jamburuthugoda, 2008; Bennetzen and Wang, 2014). The *Pol* gene is located at the 3’ end of *GAG* and encodes reverse transcriptase (RT), protease (PROT), RNase H (RH), and integrase (INT), all of which are involved in retrotransposon DNA replication and the transposition system (Gao et al., 2003). *Copia*, *Gypsy*, and *BEL–Pao* superfamilies represent LTR-RT classes according to the arrangement of internal domains (Wicker et al., 2007). There is further evidence that nonautonomous elements can retrotranspose actively or inactively (Sabot et al., 2006). Examples of nonautonomous groups include LArge Retrotransposon Derivatives (LARD) (Kalendar et al., 2004), Terminal Repeat Retrotransposons with *GAG* domain (TR-GAG) (Chaparro et al., 2015), Terminal Repeats In Miniature (TRIM) (Witte et al., 2001), and Barley RetroElement-2 (BARE-2) (Tanskanen et al., 2007).

During the replication cycle of LTR-RT, the newly inserted copy has two identical LTRs at the two ends of the element. The accumulation of mutations between the two LTRs of an LTR-RT was used to estimate the elapsed time after insertion (Zhou et al., 2021). They also show divergence caused by mutations acquired over time proportional to the age of insertion (Kijima and Innan, 2009; Neumann et al., 2019). Unequal crossovers between LTRs result in loss of internal sequence and formation of solo-LTRs (Cossu et al., 2017). In species that allow LTR-RTs accumulation, the activity of LTR-RTs is a critical factor in genome evolution, causing extremely large genome sizes (Kelly et al., 2015; Wicker et al., 2018). Genomic studies have established that LTR-RTs account for a considerable proportion of many plant genomes, including 19% of peach genome (Alseekh et al., 2020), 62% of tomato genome (Sato et al., 2012), 53% of potato genome (Diambra, 2011), and more than 70% of maize genome (Schnable et al., 2009). Tracking LTR-RT activities in plant genomes provides useful information on genome evolution and consequently gene function. The activity of LTR-RT near or within genes not only provides the raw material for structures such as centromeres and introns, but also directly alters gene function (Bennetzen and Wang, 2014; Vitte et al., 2014). LTR-RTs can influence gene regulation processes such as alternative splicing, alternative promoter control, and gene silencing (Kashkush et al., 2003; Qu et al., 2019; Yamamoto et al., 2021). Their influence on gene activity may affect the agronomic traits of various crops. According to TE-genome-wide association studies, the activity of LTR-RT is associated with several important agronomic traits, including fruit weight of tomato and width of rice grains (Akakpo et al., 2020; Alseekh et al., 2020). In addition, the activation of TE can also be triggered by environmental stress, for example, the biotic stress-responsive *Tnt1* and *Tto1* families in tobacco (Grandbastien, 2015), the heat-responsive retrotransposons *Go-on* in rice (Cho et al., 2019), the cold-responsive *Tcs* family in citrus (Butelli et al., 2012), and *ONSEN* in *Arabidopsis* (Ito et al., 2013).

Advances in genome sequencing technologies have opened new avenues for the study of LTR-RTs and for understanding their role in plant evolution. Several efforts have been made to provide a stable and well-documented LTR data resource that can be used to support current and future plant functional genomic research. Several databases for TEs in plants have been developed with general and specific research tools. These databases include Repbase (Bao et al., 2015), TREP (Wicker et al., 2002), RetrOryza (Chaparro et al., 2006), MASiVEdb (Bousios et al., 2012), MnTEdb (Ma et al., 2015), DPTEdb (Li et al., 2016), GrTEdb (Xu et al., 2017), PlaNC-TE (Pedro et al., 2018), ConTEdb (Yi et al., 2018b), SPTEdb (Yi et al., 2018a), REXdb (Neumann et al., 2019), RepetDB (Amselem et al., 2019), and CicerSpTEdb (Mokhtar et al., 2021). Although these databases provide unique and useful information about LTR-RTs in different plant genomes, they lack important details and features. Therefore, there is a need for robust, publicly available LTR-RT databases to address the growing interest in the impact of LTR-RTs on genome evolution and functionality. Such databases would be beneficial in current and future efforts to incorporate LTR-RTs annotation as a potential component for understanding the hidden dynamics of the gene regulatory system. Several studies have used such data to guide annotation in gene expression experiments (Bui and Grandbastien, 2012) or to identify retrotransposon structures such as extrachromosomal circular DNA (Mann et al., 2022).

Here, we introduce PlantLTRdb, a comprehensively designed database to expand the understanding of plant genome organization and its structural variations. PlantLTRdb provides online and searchable data resources for LTR-RT genomic information and a reliable and powerful computational service. PlantLTRdb contains detailed information on LTR-RTs in 195 plant species, both model and non-model organisms. These results are easily accessible and can be displayed using various statistical and genome-wide visualization tools. Users can download annotation files for use in advanced genomic procedures. In addition, the website provides online identification analysis for LTR-RTs *via* LTR_FINDER (Xu and Wang, 2007), which supports the standard input sequence format (FASTA).

## 2 Materials and methods

### 2.1 Plant genome data

Genome sequences of plant species and their annotations were retrieved from the NCBI database (https://www.ncbi.nlm.nih.gov/). Only genome sequences annotated and labeled as reference or representative genomes, including model and non-model plant species, were used for this analysis. The resulting dataset included 201 plant species divided into 180 Streptophyta, 18 Chlorophyta, and 3 Rhodophyta. The species name, taxonomy ID, phylum, family, assembly level, genome coverage, GenBank accession number, and genome size of all plant species can be found in **Table S1**.

### 2.2 LTR-RT identification and classification

The intact LTR-RTs were identified and classified using the EDTA pipeline (Ou et al., 2019), LTRdigest (Steinbiss et al., 2009), and TEsorter (Zhang et al., 2022) in 201 plant species. The intact LTR-RT element consists of two identical or very similar LTRs, TG-CA terms of the LTRs, and a target site duplication (TSD) (Du et al., 2010a; Dai et al., 2018). The EDTA pipeline integrates the structure-based, homology-based, and *de novo* intact LTR-RT identification tools such as LTR_FINDER (Xu and Wang, 2007), LTRharvest (Ellinghaus et al., 2008) and LTR_retriever (Ou and Jiang, 2017). The parameters of LTR_FINDER were *maximum distance between LTRs: 15000, minimum distance between LTRs: 1000, maximum LTR Length: 7000, minimum LTR Length: 100, length of exact match pairs: 20, match score: 0.85 and output format: 2.* LTRharvest parameters were *minimum LTR Length: 100, maximum LTR Length: 7000, minimum length for each TSD: 4, maximum length for each TSD: 6, motif: TGCA, maximum number of mismatches in motif: 1, similarity threshold: 85, number of nucleotides to be searched for TSDs: 10, minimum seed length for exact repeats: 20 and use sequence descriptions in GFF3 output: yes.*

LTRdigest (Steinbiss et al., 2009) was used to identify and annotate primer binding sites, polypurine tract, and tRNAs of LTR-RT elements. The tRNA sequences of the 201 plant species were retrieved from a tRNA database of plant species (Mokhtar and EL Allali, 2022). TEsorter (Zhang et al., 2022) was used to annotate coding regions and classify LTR-RTs into clades using the REXdb database. In addition, TEsorter used the 80-80-80 (identity-coverage-length) unified classification system proposed by Wicker et al. (2007) to classify identified elements. To assess the quality of assembled repetitive sequences, the LTR Assembly Index (LAI) (Ou et al., 2018) was estimated using LAI within LTR_retriever (v2.9.0) with default parameters.

**Figure 1:**
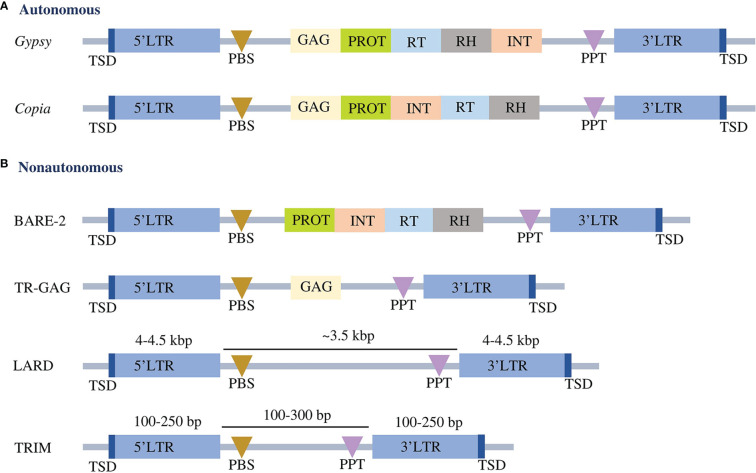
Conserved structures of autonomous **(A)** and nonautonomous **(B)** LTR-RTs. LTR refers to long terminal repeats. TSD is the target site duplication. PBS represents primer binding site, GAG represents capsid proteins, PROT represents protease, RT represents reverse transcriptase, RH represents RNase H, INT represents integrase, and PPT represents polypurine tract. The structures are not drawn to scale.

Classification of the identified LTR-RTs into putative autonomous and nonautonomous elements was based on the complete structure of the elements. Elements with a complete structure of LTR-RT were classified as autonomous, whereas incomplete elements were considered nonautonomous. The structures of autonomous *Copia* and *Gypsy* were classified using the domain orders TSD-LTR-PBS-GAG-PROT-INT-RT-RH-PPT-LTR-TSD and TSD-LTR-PBS-GAG-PROT-RT-RH-INT-PPT-LTR-TSD, respectively. Nonautonomous elements contain LARD, TR-GAG, TRIM and BARE-2, classified according to the criteria presented by Kalendar et al. (2004); Chaparro et al. (2015), Witte et al. (2001) and Tanskanen et al. (2007), respectively (**Figure 1**). The intact LTR-RT elements that were not subject to the previous conditions and defined as *Copia* or *Gypsy* were classified as nonautonomous *Copia* and nonautonomous *Gypsy*, respectively. The unknown element is defined as an intact LTR-RT that contains PBS and PPT or has lost one or both of these elements and does not contain any of the *GAG* and *Pol* domains.

LTR-RT insertion age was determined only for intact LTR-RT elements. A comparison of the 5’ and 3’ semi-identical LTRs for each LTR-RT element was used to calculate the insertion age. This comparative analysis was carried out using ClustalW (Thompson et al., 2003) to obtain a local alignment of the two LTRs. The estimation of the insertion age based on the method of Tajima and Nei (Tajima and Nei, 1984) and the Kimura-2 parameter model (Kimura, 1980) was performed with REANNOTATE (Pereira, 2008). Nucleotide substitutions per site (K) between LTRs were estimated using the Kimura-2 parameter model. The estimated age was calculated using the formula T= K/2r. The evolutionary rate (r) of 1.3×10^–8^ substitutions per site per year was used for grass plants (Kimura, 1980; Ma and Bennetzen, 2004), whereas a substitution rate of 1.5×10^–8^ was used for other species as reported in the literature (Koch et al., 2000; Gonzalez and Deyholos, 2012; Marcon et al., 2015). Here, we used a substitution rate of 1.5×10^–8^ for plants other than grasses because an average substitution rate is not available for many plants.

**Figure 2:**
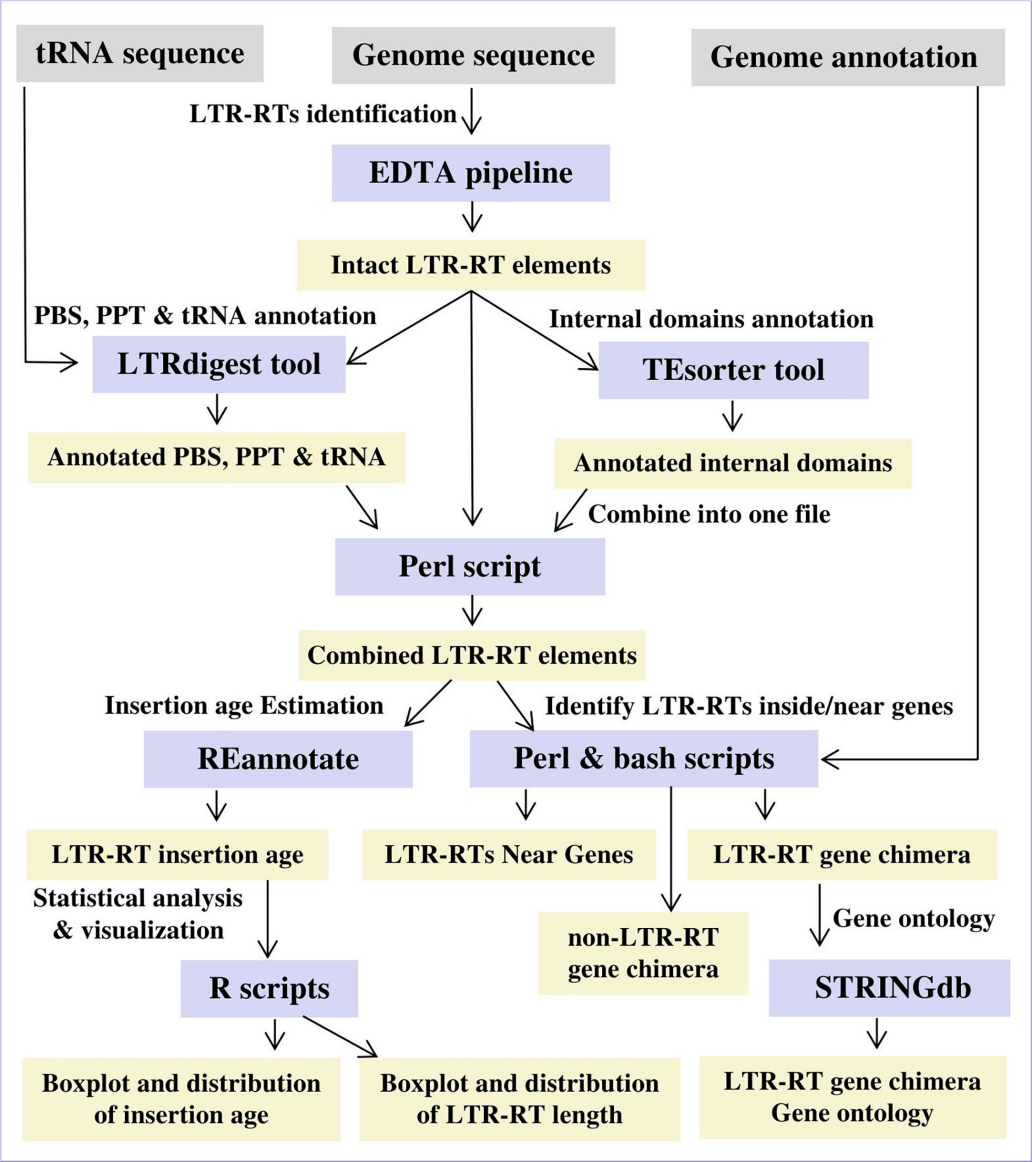
The workflow and procedure for identifying and characterizing LTR-RT in 201 plant species.

Based on genomic position, identified LTR-RTs were classified into LTR-RT-gene chimeras by comparing the start and end coordinates of genes and LRT-RTs within the genome. LTR-RT was considered an LTR-RT-gene chimera if it was within the gene start and end coordinates. A gene ontology was assigned to all genes that contained LTR-RT elements or were in close proximity using STRINGdb (Szklarczyk et al., 2015). Gene enrichment analysis was performed using *Arabidopsis thaliana* and *Medicago truncatula* as model plants. **Figure 2** shows the workflow and procedure used in the data analysis. Statistical correlations between plant genome size, LTR-RT length, and insertion age were performed for all diploid plant species with LAI ≥10. LAI is the ratio of the length of intact LTR-RTs to the total LTR length (Ou et al., 2018). Scripts used for data analysis are available on GitHub for public use at https://github.com/agc-bioinformatics/PlantLTRdb.

### 2.3 Database development

The PlantLTRdb was created as a hub and interactive web interface using a variety of programming languages including Perl, Python, R, MongoDB, PHP, CSS, HTML, and JavaScript. In addition, PlantLTRdb includes an implementation of a simple interface for the software LTR_FINDER (Xu and Wang, 2007). The easy-to-use LTR_FINDER interface allows users to identify LTR-RT elements using the standard input sequence format (FASTA). PlantLTRdb is hosted on a server with 32 GB of memory, 16-core CPUs, and a 10 TB disk; running Linux 5.4.0-89-generic x86 64, Apache 2.4, MongoDB and PHP 7.4.3. The online tools require Python (v3.8.10), Perl (v5.30.0), and R (v4.1.2). HTCondor (v9.5.0) is used to manage and schedule submitted tasks and processes. The frontend of the website is built using the Vue (3.2) framework, while the backend is built using Laravel (8.75). Users can select, filter, and visualize available LTR-RT information based on the processed plant species using several online tools. Several tools were used for data visualization, including JBrowse (Buels et al., 2016), ggplot2 (R package), and Google Charts (https://developers.google.com/chart).

## 3 Results and discussion

### 3.1 Identification and classification of LTR-RT elements

In the last decade, the complete genome sequences of hundreds of plant species have been published (Mokhtar and Atia, 2018). Access to these extensive data has paved the way for the study of LTR-RTs at the genome level. Over the past decade, LTR-RTs from several plant species have been identified and classified using homology, structural, and *de novo* investigation methods (Wicker et al., 2018; Yi et al., 2018b; Neumann et al., 2019; Mokhtar et al., 2021; Zhou et al., 2021). The development of a unified, well-maintained, effective resource for plant LTR-RT is a prerequisite to support progress in understanding the functional effects of these factors on genomic structure and functionality. Ou et al. (2018) reported that more intact LTR-RTs were identified from complete genome assemblies compared with draft genomes. In addition, other reports indicated a correlation between sequencing technique and the number of intact LTR-RTs detected (Al-Dous et al., 2011; Jiao et al., 2017; Ou et al., 2018). These reports suggest that more intact LTR-RTs are detected from continuous genome assembly. In the current study, we used only annotated genomes because annotation is required to identify LTR-RT-gene chimeras and LTR-RT nearby genes.

We used established and validated LTR-RT tools to create a workflow for the identification and classification of LTR-RTs in different plant species (**Figures 1**, **2**). The resulting data were used to create a user-friendly public resource for intact LTR-RT in plants. The PlantLTRdb was developed by processing the entire genomic sequences of 201 plant species, totaling 150.18 Gbp. These sequences represent genomic data from 3,079,469 pseudomolecules/scaffolds. However, 6 genomes, including *Chloropicon primus*, *Cyanidioschyzon merolae*, *Galdieria sulphuraria*, *Genlisea aurea*, *Micromonas commoda*, and *Monoraphidium neglectum*, failed the EDTA filtering step and no intact LTR-RTs were found. These genomes were excluded from the analysis and only 195 plant species that passed the filtering step were used for further analysis.

**Figure 3:**
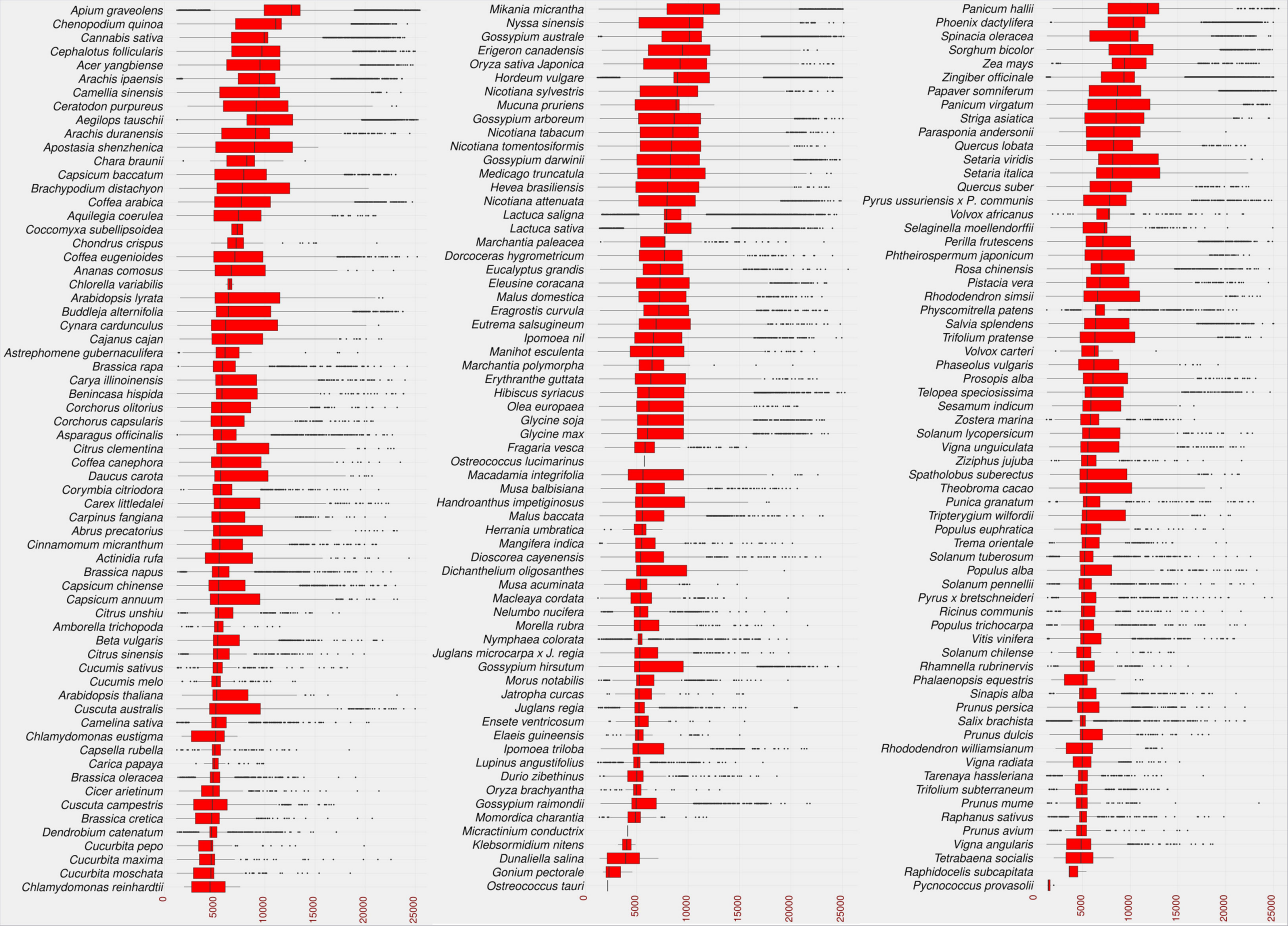
Statistical overview of the LTR-RT length by base pair. The boxplot of the LTR-RT length in the studied plant species. Species sorted in descending order by the median value. The LTR-RT lengths are shown in bp scale (x-axis).

As a result, 2,722,415 LTR candidates were identified in the studied species. The identified LTRs were filtered based on the intact LTR-RT structure (TSD-LTR-[internal sequence]-LTR-TSD) and candidates with missing components were excluded from further analysis. Only 528,891 candidates passed filtering and had intact LTR-RT structures. The candidates that include nested LTRs or other TEs insertions were excluded using LTR_retriever module 6 (Ou et al., 2018). The remaining 520,194 elements were then annotated using LTRdigest and TEsorter to identify PBS, PPT, *GAG*, and *Pol* regions and classify them into lineages. Table S2 shows the lineages and their corresponding totals in the database. Based on the structure of the autonomous LTR-RT, the identified intact LTR-RTs were classified into putative 29,462 autonomous and 490,732 non-autonomous LTR-RTs. The 29,462 autonomous LTR-RTs include 10,286 from the *Gypsy* superfamily and 19,176 from the *Copia* superfamily. Further analyses were performed to classify non-autonomous elements using the criteria presented by Witte et al. (2001); Kalendar et al. (2004); Tanskanen et al. (2007); Chaparro et al. (2015). In addition, incomplete *Copia* and *Gypsy* elements were classified as nonautonomous. All non-autonomous elements not subject to any of the above structures were defined as unknown elements. Based on these criteria, 490,732 nonautonomous elements were divided into 224,906 *Gypsy*, 218,414 *Copia*, 1,768 BARE-2, 3,147 TR-GAG, and 42,497 unknown, while LARD and TRIM elements were not present (Table S3). The number of LTR-RTs detected ranged from 1 (*Micractinium conductrix*) to 33,245 (*Aegilops tauschii*). After excluding outliers by boxplot analysis of LTR-RT length for all 195 plant species, the results showed that the minimum, first quartile, median, third quartile, and maximum lengths were 1,140, 5,398, 8,273, 11,061, and 19,555, respectively (**Figure 3**). The analysis also showed that most plant species had a wide range of LTR-RT lengths (**Figure S1**).

**Figure 4:**
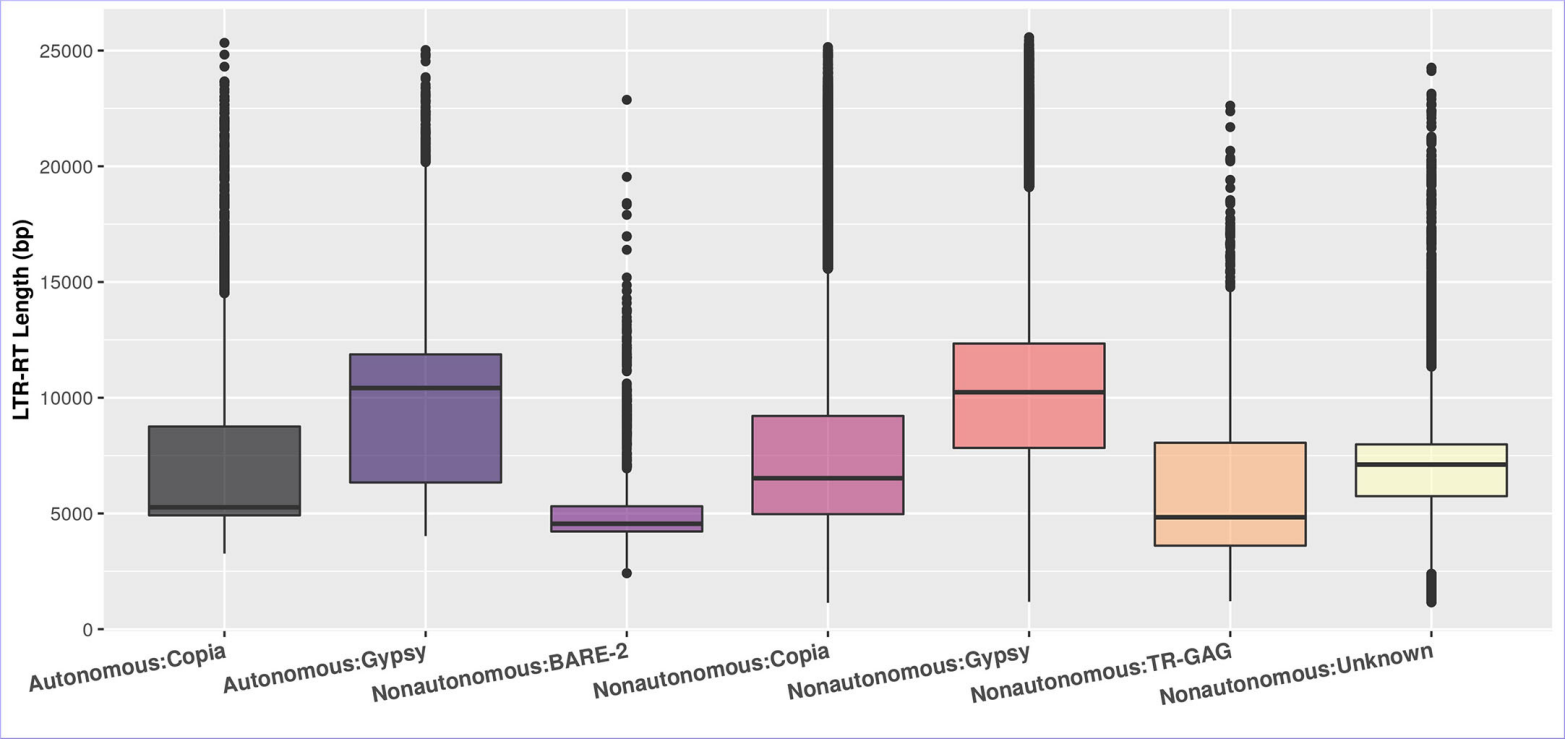
The boxplot of the LTR-RT length in the studied plant species of the autonomous and nonautonomous elements. The LTR-RT lengths are shown in bp scale (Y-axis).

The differences in the length of LTR-RT are primarily due to divergence in the size of LTR and the existence and size of spacer regions between internal domains rather than *GAG/Pol* coding regions (Zhou et al., 2017). **Figure 4** shows that after excluding outliers by boxplot analysis, the first quartile, median, and third quartile of the autonomous LTR-RTs were 4,920, 5,267, 8,757 bp for *Copia* and 6,337, 10,420, 11,873 bp for *Gypsy*, respectively. For nonautonomous LTR-RTs, the first quartile, median, and third quartile were 4,971, 6,522, 9,213 bp for *Copia*, 7,832, 10,237, 12,340 bp for *Gypsy*, 3,608, 4,837, 8,054 bp for TR-GAG, 4,220, 4,555, 5,310 bp for BARE-2 and 5,746, 7,112, 7,985 bp for unknown, respectively. The first quartile, median, third quartile, minimum, and maximum of LTR-RT length for the 195 plant species were listed in Table S4. In the present study, based on the parameters used to identify LTR-RTs, and without excluding outliers by boxplot analysis, the length of autonomous and nonautonomous *Copia* elements ranged from 1,140 to 25,333 bp, whereas the length of *Gypsy* ranged from 1,182 to 25,575 bp. This is consistent with previous studies by Du et al. (2010b); Ma et al. (2019); Neumann et al. (2019); Li et al. (2022), which found that a number of *Gypsy* elements were smaller than 4kb and a number of *Copia* elements were larger than 15kb. For example, Li et al. (2022) examined LTR-RTs of 16 *Cucurbitaceae* species and reported that the length of LTR-RTs ranged from 1,173 to 28,350 bp. Boxplot analysis showed *Gypsy* elements smaller than 4kb and *Copia* elements larger than 15kb.

**Figure 5:**
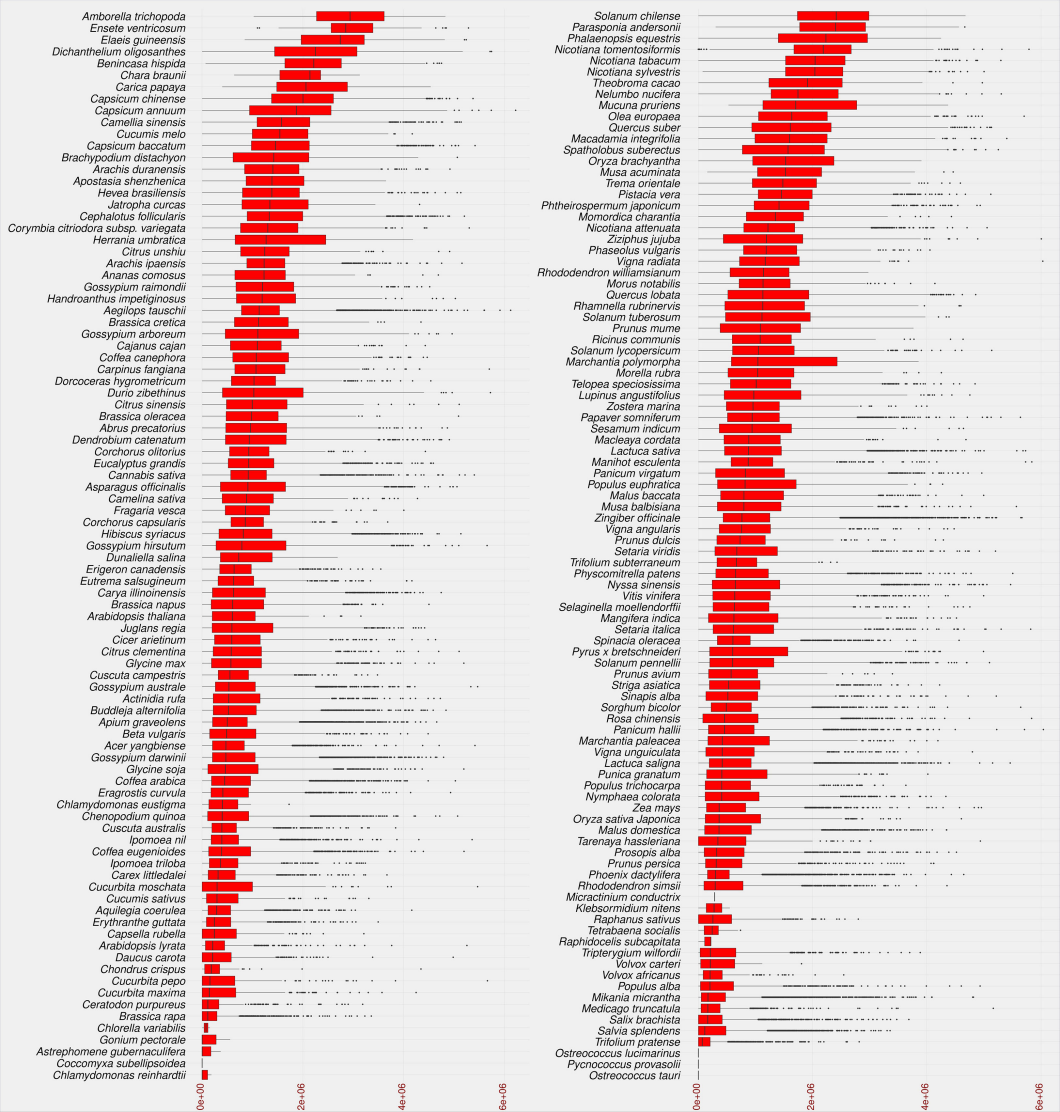
Statistical overview of the age of LTR-RT insertion in the studied plant species using boxplot analysis. Species are sorted in descending order by median value. Values for age of LTR-RT insertion are in years (x-axis).

The insertion age of the plant species studied reflects the evolutionary rate associated with the uniqueness of their genomic content. This evolutionary difference can help researchers understand the relationship between genetic and phenotypic variation (Barghini et al., 2014). Recently, TE-family has proven particularly useful in understanding the evolutionary mechanisms involved in species divergence (Liu and Yang, 2014). Transposition of LTR-RTs results in identical sequences of 5’ and 3’ LTRs, and the accumulation of nucleotide substitutions/divergences between the two arms of LTR-RTs is used to calculate the insertion age (da Costa et al., 2019; Mokhtar et al., 2021). **Figure 5** shows that the maximum assumed age after exclusion of outliers by boxplot analysis was 5.1 million years (MY) for *Dichanthelium oligosanthes*. Several plant species have a high rate of young LTR-RTs in their genome, such as *Phoenix dactylifera*, *Cucumis sativus*, *Arabidopsis lyrata*, *Daucus carota*, *Medicago truncatula*, and *Brassica rapa*. In addition, other plant species show a homogeneous collection of LTR-RTs with different insertion ages, such as *Solanum chilense*, *Carica papaya*, *Theobroma cacao*, *Capsicum annuum*, and *Mucuna pruriens*. Our results are consistent with previous findings for some of these plant species. According to a previous analysis, most LTR-RTs identified in *Medicago truncatula* are relatively recent and were inserted in the last 0.52 MY, with possibly more than 10 million bp lost due to deletion of LTR elements and removal of full-length structures (Wang and Liu, 2008). Ibrahim et al. (2021) examined LTR-RTs in numerous palm genomes and concluded that the *Elaeis guineensis* genome has undergone several LTR-RT events with different temporal patterns of transposition activity. In our study, the first quartile of insertion age in *Ensete ventricosum*, *Amborella trichopoda* and *Elaeis guineensis* shows the highest values (1.9-2.5 MY), while the maximum third quartile has the lowest values in *Chlamydomonas reinhardtii*, *Chlorella variabilis*, *Astrephomene gubernaculifera*, *Trifolium pratense*, and *Raphidocelis subcapitata* (0.11-0.21 MY). The third quartile of LTR-RT insertion age ranges from 0.11 MY in *Chlamydomonas reinhardtii* to 3.6 MY in *Amborella trichopoda*.

The different distribution of LTR-RT insertion age among the studied species suggests that there is a relationship between the overall insertion age and the insertion age of each LTR-RT type. Most plant species with a high first quartile of insertion age, such as *Elaeis guineensis*, have a wide range of insertion age values, whereas those with a high rate of young LTR-RTs have a narrow range of insertion age. On the other hand, some species such as *Capsicum annuum* and *Ziziphus jujuba* have narrow and wide ranges of insertion age in different parts of the LTR-RT distribution (**Figure S2**).

**Figure 6:**
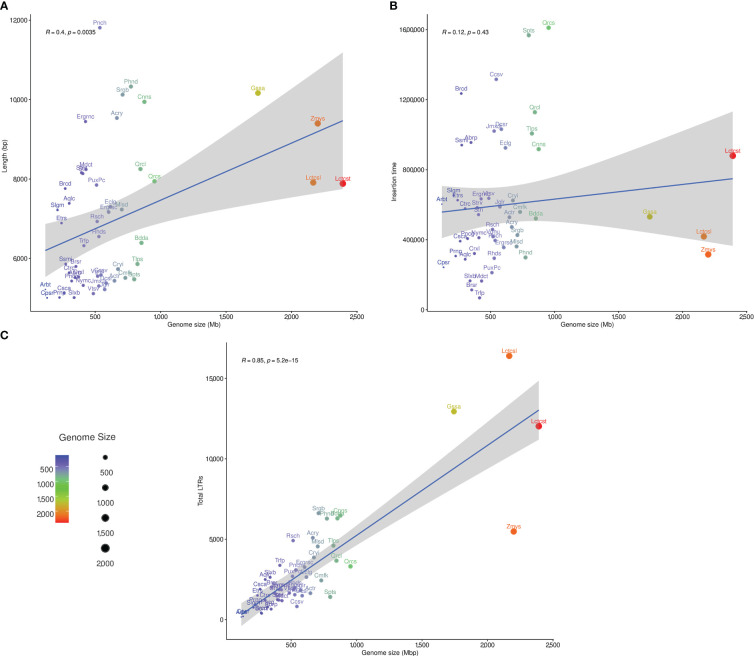
The statistical correlation between plant genome size, and LTR-RT length **(A)**, insertion age **(B)**, and total number of LTR-RT **(C)**.

The LAI was estimated for studied plant genomes using the LAI program within LTR_retriever (Ou et al., 2018). Only the diploid plant species with LAI ratio greater than 10 (50 species) were subjected to correlation analysis between genome size, LTR-RT estimated insertion age and LTR-RT length. The correlation between genome size and LTR-RT length was 0.4 (R), with a p-value of 0.0035, indicating that it was significant. This suggests that although there is a clear correlation between genome size and intact LTR-RT length, the effect of genome size on length is weak (**Figure 6A**). The correlation between the genome size and LTR-RT insertion age was 0.12 (R), with a p-value of 0.43 indicating a non-significant association. This suggests that there is no relationship between genome size and insertion age. It also suggests that genome size has little or no effect on LTR-RT insertion age (**Figure 6B**). Some plant species, such as *Amborella trichopoda* and *Elaeis guineensis* have medium or small genome sizes with a high LTR-RT insertion age. The correlation between total LTR-RTs and genome size is 0.85 (R), with a p-value of 5.2×10^–15^, showing a strong positive correlation between them (**Figure 6C**).

LTR-RT transposition can affect the expression of both housed LTR-RT-gene chimeras and nearby genes. LTR-RTs influence genes through the processes of movement, duplication, and recombination constructing or modifying gene structure (Zhao et al., 2016). Further analysis was performed on the plant species studied to identify LTR-RTs located within or near genes. In several plant species, the interaction of LTR-RT activity in genic regions could result in a hybrid of LTR-RT-gene structures or LTR-RT-gene chimeras (Jiang et al., 2004; Wang et al., 2006). A total of 37,206 LTR-RTs were classified as LTR-RT-gene chimeras in all species studied, and 11,844 LTR-RTs were found within pseudo-genes (Table S5). In addition, 300,613 genes were up to 10 Kbp from LTR-RTs. Table S5 shows that of the 300,613 genes, 50,026 were located up to 1 Kbps away and 250,587 were within 1 to 10 Kbp. The *Copia* superfamily, found within genes, was more prevalent than *Gypsy* elements in the current study, consistent with previous studies in some plant species (Bennetzen, 1996; Rossi et al., 2001; Lockton and Gaut, 2009; Mokhtar et al., 2021). Using the gene ontology of two different model plant species, gene enrichment analysis was performed for genes located within or near LTR-RTs in the plant genomes studied. Gene ontologies such as binding, cell membrane, and catalytic activity were highly enriched in LTR-RTs associated genes. The high frequency of genes associated with catalytic activity and binding may be related to the biological activity of LTR-RTs within the plant genome to promote gene expression, such as those associated with stress response (Bui and Grandbastien, 2012).

### 3.2 Technical validation

To verify the quality of the identified intact LTR-RTs in the current study, a manually curated LTR-RTs library of rice (*Oryza sativa. ssp. japonica*) was used for comparison with our *Oryza sativa* dataset. *Oryza sativa* was selected for comparison because its genome sequence is well structured and arranged in chromosomes and has a high LAI score of 22.41. The curated rice library was presented in a previous study by Ou and Jiang (2017) and included 897 LTR-RTs elements. TEsorter (Zhang et al., 2022) was used to annotate the *GAG*- and *Pol* domains of this library (897 LTR-RTs) using the REXdb database (Neumann et al., 2019) based on a unified LTR-RTs classification rule (80-80-80) as proposed by Wicker et al. (2007). Of the 897 LTR-RTs, 242 elements have a complete *GAG*- and *Pol* domains, which were used for comparison with the currently identified LTR-RTs of *Oryza sativa* using the OrthoFinder tool (Emms and Kelly, 2019). In our results, the LTR-RTs dataset of *Oryza sativa* contains 1,496 LTR-RT elements divided into 54 autonomous and 1,442 nonautonomous. Using OrthoFinder, 1,114 elements were assigned to the curated library ortho-groups (Table S6). The remaining 382 elements also have strong evidence as they include 204 elements that have all necessary domains for their transposition, 17 contain four domains, six contain three domains, 25 contain two domains, 97 contain one domain, and 33 elements are unknown (Table S6). This comparison verifies the reliability of the identified LTR-RTs in the current investigation.

### 3.3 PlantLTRdb as a resource for intact LTR-RTs in plants

**Figure 7:**
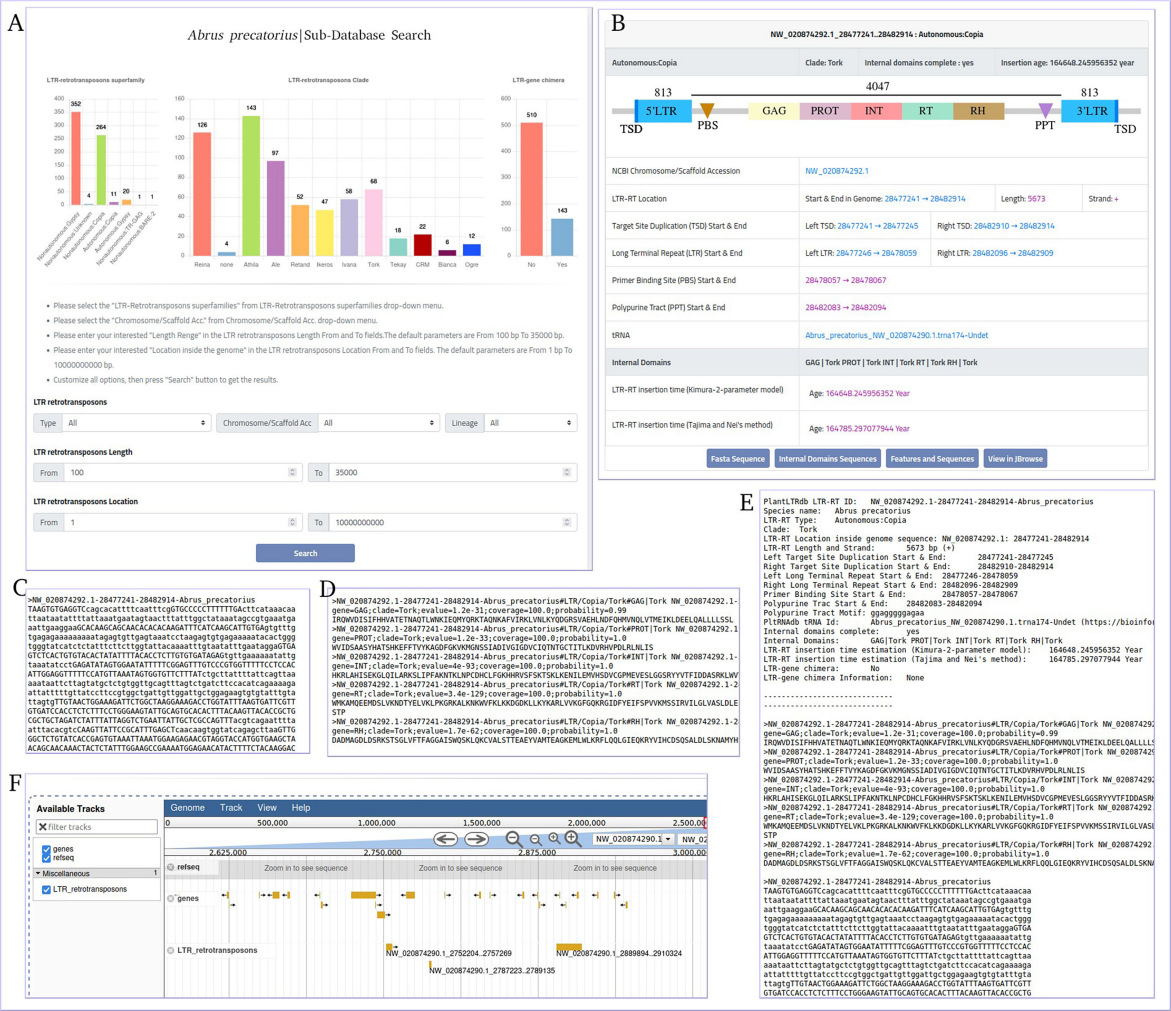
*Abrus precatorius* sub-database search page as an example of PlantLTRdb search. **(A)** general search page, **(B)** example of general search results, **(C)** LTR-RT FASTA sequence, **(D)** internal domains FASTA sequence, **(E)** LTR-RT features, and **(F)** JBrowse example.

The data generated by the LTR-RT analysis workflow during the processing of 195 plant genomes were used to build a flexible, efficient, and well-maintained database of LTR-RT elements and associated information in the plant species studied (**Figure S3**). Our plant LTR-RTs database (PlantLTRdb) is accessible through an easy-to-use web interface. Through the public website portal, users can search, visualize, BLAST, and analyze plant LTR-RT elements. The PlantLTRdb search dropdown menu provides users with access to two separate search pages. The first allows a general search for LTR-RTs from all plant species studied, and the second allows a search for detected LTR-RT-gene chimeras and nearby genes. On the LTR-RTs general search page, users can view bar charts summarizing the number of identified LTR-RTs in the species searched. In addition, several search options are available, including searching by LTR-RT superfamily, pseudomolecules/scaffolds, LTR-RT length, LTR-RT position in the genome, and searching by all of the above criteria (**Figure 7A**). All results are displayed on a separate page with additional information about LTR-RTs including, NCBI accession, LTR-RT position in genome, length, type, target site duplication, long terminal repeat, primer binding site, polypurine tract, tRNA, internal domains, LTR-RT insertion age, JBrowse link and download buttons for LTR-RT FASTA file, internal domains FASTA file, and LTR-RT features (**Figures 7B–F**).

**Figure 8:**
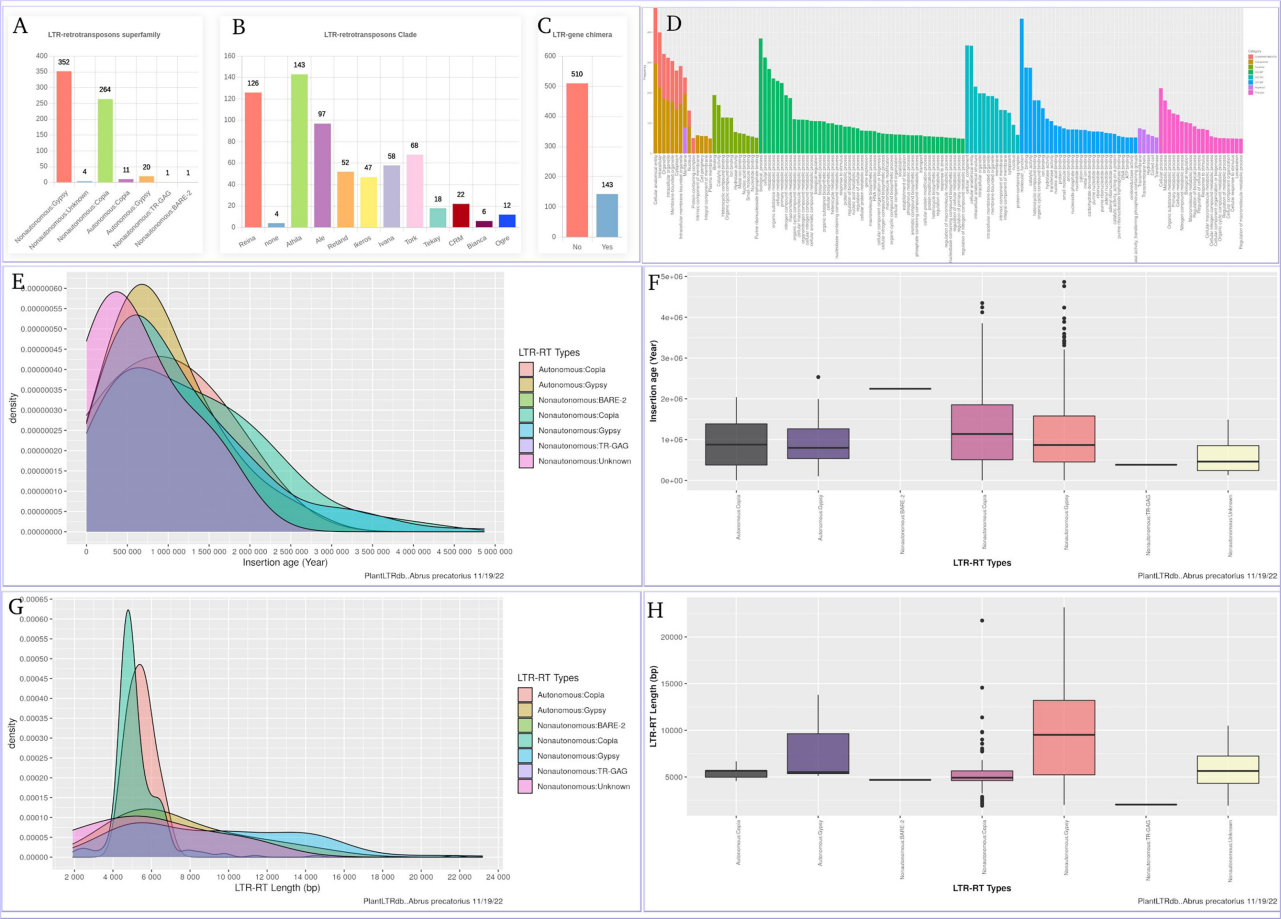
An example of the statistics page using Abrus precatorius. **(A)** LTR-RTs superfamily statistics, **(B)** LTR-RTs clade, **(C)** LTR-RT-gene chimera statistics, **(D)** Gene ontology, **(E, F)** statistical overview of the LTR insertion age, **(G, H)** statistical overview of the LTR-RT length by bp.

The LTR-RT gene interaction search page provides users with bar charts summarizing the number of detected LTR-RT-gene chimeras and neighboring genes within the searched species. In addition, several search options are available, including searching by LTR-RT superfamily, gene category, NCBI gene ID/locus tag, and/or protein ID. Generated results include NCBI gene ID, gene start and end, gene description, distance between LTR-RT and gene, protein ID, gene ontology, superfamily, LTR-RT position in genome, length, type, target site duplication, long terminal repeat, primer binding site, polypurine tract, tRNA, internal domains, LTR-RT insertion age, JBrowse link and download buttons for LTR-RT FASTA sequence, internal domains FASTA sequence and LTR-RT features (**Figure S4**). Users can visualize LTR-RTs at the genome level with JBrowse, which is integrated into PlantLTRdb. The JBrowse page displays information about the selected plant species, such as the reference genome sequence, genome annotations (genes), and any LTR-RT coordinates that have been identified. Users can view the details of LTR-RT elements and evaluate LTR-RT nearby genes. The statistics page provides the user with interactive graphs for the LTR-RTs superfamily statistics, LTR-RT-gene chimera statistics, LTR-RTs clade, statistical overview of the LTR insertion age, the LTR-RT length by bp, and the gene ontology of the LTR-RT-gene chimeras and nearby genes for each plant species. Several statistical plots are generated to cover all aspects of the results (**Figure 8**).

PlantLTRdb provides powerful tools for searching for specific LTR-RT elements in processed genomic data or user-supplied data. Our online BLASTN allows users to align their LTR-RT sequences against the LTR-RTs of the specified plant species. Results from this tool include the known BLAST results and a link to similar LTR-RT details stored in PlantLTRdb, such as pseudomolecules/scaffold accession, LTR-RT genomic position, length, insertion age, sequence, and JBrowse profile link. In addition, an online version of LTR_Finder (Xu and Wang, 2007) has been integrated into the PlantLTRdb platform. This tool allows users to explore LTR-RTs in their own genomic data. The LTR_FINDER webserver (http://tlife.fudan.edu.cn/ltr_finder/) has been decommissioned for unknown reasons, and only the local version is available for use. LTR_FINDER will help many researchers to investigate LTR-RT elements using their genomic data.

### 3.4 Comparison with other TEs databases

Existing databases provide useful information on LTR-RTs in various plant genomes, but they still lack important details and features (**Table 1**). For example, Repbase (Bao et al., 2015) is a dataset for eukaryotic repetitive sequences including LTR-RTs. The LTR-RTs have been classified into superfamilies and lineages, but details of internal structure are lacking. In addition, Repbase requires a subscription for access. REXdb (Neumann et al., 2019) contains LTR-RTs protein domains from 80 species. The LTR-RT elements are detected using LTR_FINDER and the data are not available as a stand-alone database, but can only be downloaded from the RepeatExplorer website. In addition, PlaNC-TE (Pedro et al., 2018) contains only overlapping regions between TEs and non-coding RNAs (ncRNAs) in 40 plant species. RepetDB (Amselem et al., 2019) contains TEs for 23 species. MnTEdb (Ma et al., 2015), DPTEdb (Li et al., 2016), ConTEdb (Yi et al., 2018b), SPTEdb (Yi et al., 2018a), and CicerSpTEdb (Mokhtar et al., 2021) are databases containing only TEs from 1 to 8 genomes, while MASiVEdb (Bousios et al., 2012), GrTEdb (Xu et al., 2017), and RetrOryza (Chaparro et al., 2006) are no longer available.

**Table 1:** Comparison of online TE databases based on the number of species and their LTR-RT related features.

Database	Species	Search	Insertion time	LTR-RT in/near genes	Buit-in tools	Availability
PlantLTRdb	195	✔	✔	✔	JBrowse, Blast, LTR Finde	https://bioinformatics.um6p.ma/PlantLTRdb
REXdb	80	×	×	×	–	http://repeatexplorer.org/?pageid=918
TREP	60	✔	×	×	Blast	http://botserv2.uzh.ch/kelldata/trep-db
PlaNC-TE	40	✔	×	×	JBrowse	http://planc-te.cp.utfpr.edu.br/
RepetDB	23	✔	×	×	Blast	https://urgi.versailles.inra.fr/repetdb/begin.do
MASiVEdb	11	✔	×	×	–	No longer Available
DPTEdb	8	✔	×	×	JBrowse, Blast, GetORF, Hmm, Cut sequence	http://genedenovoweb.ticp.net:81/DPTEdb
CicerSpTEdb	3	✔	×	✔	JBrowse	http://cicersptedb.easyomics.org
ConTEdb	3	✔	×	×	JBrowse, Blast, GetORF, Hmm, Cut sequence	http://genedenovoweb.ticp.net:81/conTEdb
SPTEdb	3	✔	×	×	JBrowse, Blast, GetORF, Hmm, Cut sequence	http://genedenovoweb.ticp.net:81/SPTEdb
GrTEdb	1	✔	×	×	–	No longer Available
RetrOryza	1	✔	×	×	–	No longer Available
MnTEdb	1	✔	×	×	JBrowse, Blast, GetORF, Hmm, Cut sequence	https://morus.swu.edu.cn/mntedb/

PlantRep (Luo et al., 2022), InpactorDB (Orozco-Arias et al., 2021), and APTEdb (Pedro et al., 2021) databases were recently published and contain TEs of 459, 195, and 67 plant species, respectively. Although these databases provide useful information on plant LTRs, they lack important features such as the classification of LTR as intact LTR-RT and into autonomous and nonautonomous elements. InpactorDB, for example, has only a single function, which is to search using a set of parameters, and does not provide visualization, bulk downloading, or built-in tools for manipulating the data. APTEdb contains a small number of plant genomes and only allows downloading the LTRs of each genome as gff and fasta files. PlantRep contains genomes that are not annotated and does not include tools such as visualization and searching. Because gene annotation information is limited, users of the existing databases may not be able to understand the impact of LTR-RTs on the plant genome and their association with specific genes or biological processes. In addition to limitations in the data, some databases have limited features and are rarely maintained and updated. Finally, most databases do not include an online LTR-RT identification tool that can be used to analyze user-specific data. Such tools would benefit those attempting to annotate newly sequenced genomic data. Compared to previously published LTR-RT databases, PlantLTRdb has unique features that can contribute to the understanding of the structural variations and organization of LTR-RTs in the genome.

## 4 Conclusions and future directions

PlantLTRdb is a hub portal of LTR-RTs in plant species. For all plant species studied, various analyzes were performed to identify, characterize, and annotate LTR-RTs, as well as to estimate insertion ages, detect LTR-RT-gene chimeras, and determine nearby genes. The PlantLTRdb contains 520,194 intact LTR-RTs, including 29,462 autonomous and 490,732 nonautonomous LTR-RTs. In addition, the website portal allows users to search, visualize, BLAST, and analyze plant LTR-RT elements. PlantLTRdb will be continuously updated with newly annotated genomes. PlantLTRdb is an important database that can contribute to the understanding of structural variations, genome organization, and the development of LTR-RT target markers for molecular plant breeding.

## Data availability statement

The datasets presented in this study can be found in online repositories. The names of the repository/repositories and accession number(s) can be found in the article/**Supplementary Material**.

## Author contributions

MMM and AE Conceptualization, Formal analysis. MMM, AMA and AE. Data curation, Methodology, Visualization, wrote and reviewed the manuscript. AE. Supervision and Resources. All authors contributed to the article and approved the submitted version.

## References

[B1] Akakpo R. Carpentier M.-C. Ie Hsing Y. Panaud O. (2020). The impact of transposable elements on the structure, evolution and function of the rice genome. New Phytol. 226, 44–49. doi: 10.1111/nph.16356 31797393

[B2] Al-Dous E. K. George B. Al-Mahmoud M. E. Al-Jaber M. Y. Wang H. Salameh Y. M.. (2011). *De novo* genome sequencing and comparative genomics of date palm (phoenix dactylifera). Nat. Biotechnol. 29, 521–527. doi: 10.1038/nbt.1860 21623354

[B3] Alseekh S. Scossa F. Fernie A. R. (2020). Mobile transposable elements shape plant genome diversity. Trends Plant Sci. 25, 1062–1064. doi: 10.1016/j.tplants.2020.08.003 32863103

[B4] Amselem J. Cornut G. Choisne N. Alaux M. Alfama-Depauw F. Jamilloux V.. (2019). RepetDB: a unified resource for transposable element references. Mobile DNA 10, 1–8. doi: 10.1186/s13100-019-0150-y 30719103 PMC6350395

[B5] Bao W. Kojima K. K. Kohany O. (2015). Repbase update, a database of repetitive elements in eukaryotic genomes. Mobile DNA 6, 1–6. doi: 10.1186/s13100-015-0041-9 PMC4455052 26045719

[B6] Barghini E. Natali L. Giordani T. Cossu R. M. Scalabrin S. Cattonaro F.. (2014). LTR Retrotransposon dynamics in the evolution of the olive (Olea europaea) genome. DNA Res. 22, 91–100. doi: 10.1093/dnares/dsu042 25428895 PMC4379980

[B7] Bennetzen J. L. (1996). The contributions of retroelements to plant genome organization, function and evolution. Trends Microbiol. 4, 347–353. doi: 10.1016/0966-842X(96)10042-1 8885169

[B8] Bennetzen J. L. Wang H. (2014). The contributions of transposable elements to the structure, function, and evolution of plant genomes. Annu. Rev. Plant Biol. 65, 505–530. doi: 10.1146/annurev-arplant-050213-035811 24579996

[B9] Bousios A. Minga E. Kalitsou N. Pantermali M. Tsaballa A. Darzentas N. (2012). MASiVEdb: the sirevirus plant retrotransposon database. BMC Genomics 13, 158. doi: 10.1186/1471-2164-13-158 22545773 PMC3414828

[B10] Buels R. Yao E. Diesh C. M. Hayes R. D. Munoz-Torres M. Helt G.. (2016). JBrowse: a dynamic web platform for genome visualization and analysis. Genome Biol. 17, 66. doi: 10.1186/s13059-016-0924-1 27072794 PMC4830012

[B11] Bui Q. T. Grandbastien M.-A. (2012). LTR Retrotransposons as Controlling Elements of Genome Response to Stress? In: Grandbastien M. A. Casacuberta J. (eds) Plant Transposable Elements. Topics in Current Genetics, vol 24. Springer, Berlin, Heidelberg. doi: 10.1007/978-3-642-31842-9_14

[B12] Butelli E. Licciardello C. Zhang Y. Liu J. Mackay S. Bailey P.. (2012). Retrotransposons control fruit-specific, cold-dependent accumulation of anthocyanins in blood oranges. Plant Cell 24, 1242–1255. doi: 10.1105/tpc.111.095232 22427337 PMC3336134

[B13] Chaparro C. Gayraud T. de Souza R. F. Domingues D. S. Akaffou S. Laforga Vanzela A. L.. (2015). Terminal-repeat retrotransposons with gag domain in plant genomes: a new testimony on the complex world of transposable elements. Genome Biol. Evol. 7, 493–504. doi: 10.1093/gbe/evv001 25573958 PMC4350172

[B14] Chaparro C. Guyot R. Zuccolo A. Piegu B. Panaud O. (2006). RetrOryza: a database of the rice LTR-retrotransposons. Nucleic Acids Res. 35, D66–D70. doi: 10.1093/nar/gkl780 17071960 PMC1635335

[B15] Cho J. Benoit M. Catoni M. Drost H.-G. Brestovitsky A. Oosterbeek M.. (2019). Sensitive detection of pre-integration intermediates of long terminal repeat retrotransposons in crop plants. Nat. Plants 5, 26–33. doi: 10.1038/s41477-018-0320-9 30531940 PMC6366555

[B16] Cossu R. M. Casola C. Giacomello S. Vidalis A. Scofield D. G. Zuccolo A. (2017). Ltr retrotransposons show low levels of unequal recombination and high rates of intraelement gene conversion in large plant genomes. Genome Biol. Evol. 9, 3449–3462. doi: 10.1093/gbe/evx260 29228262 PMC5751070

[B17] da Costa Z. P. Cauz-Santos L. A. Ragagnin G. T. Van Sluys M.-A. Dornelas M. C. Berges H.. (2019). Transposable element discovery and characterization of LTR-retrotransposon evolutionary lineages in the tropical fruit species passiflora edulis. Mol. Biol. Rep. 46, 6117–6133. doi: 10.1007/s11033-019-05047-4 31549373

[B18] Dai X. Wang H. Zhou H. Wang L. Dvořak J. Bennetzen J. L.. (2018). Birth and death of ltr-retrotransposons in aegilops tauschii. Genetics 210, 1039–1051. doi: 10.1534/genetics.118.301198 30158124 PMC6218219

[B19] Diambra L. A. (2011). Genome sequence and analysis of the tuber crop potato. Nature 475, 189–195. doi: 10.1038/nature10158 21743474

[B20] Du J. Tian Z. Bowen N. J. Schmutz J. Shoemaker R. C. Ma J. (2010a). Bifurcation and enhancement of autonomous-nonautonomous retrotransposon partnership through ltr swapping in soybean. Plant Cell 22 (1), 48–61. doi: 10.1105/tpc.109.068775 20081112 PMC2828711

[B21] Du J. Tian Z. Hans C. S. Laten H. M. Cannon S. B. Jackson S. A.. (2010b). Evolutionary conservation, diversity and specificity of ltr-retrotransposons in flowering plants: Insights from genome wide analysis and multi-specific comparison. Plant J. 63 (4), 584–598. doi: 10.1111/j.1365-313X.2010.04263.x 20525006

[B22] Eickbush T. H. Jamburuthugoda V. K. (2008). The diversity of retrotransposons and the properties of their reverse transcriptases. Virus Res. 134, 221–234. doi: 10.1016/j.virusres.2007.12.010 18261821 PMC2695964

[B23] Ellinghaus D. Kurtz S. Willhoeft U. (2008). LTRharvest, an efficient and flexible software for *de novo* detection of LTR retrotransposons. BMC Bioinf. 9, 1–14. doi: 10.1186/1471-2105-9-18 PMC2253517 18194517

[B24] Emms D. M. Kelly S. (2019). Orthofinder: phylogenetic orthology inference for comparative genomics. Genome Biol. 20, 1–14. doi: 10.1186/s13059-019-1832-y 31727128 PMC6857279

[B25] Gao X. Havecker E. R. Baranov P. V. Atkins J. F. Voytas D. F. (2003). Translational recoding signals between gag and pol in diverse LTR retrotransposons. RNA 9, 1422–1430. doi: 10.1261/rna.5105503 14623998 PMC1370496

[B26] Gonzalez L. G. Deyholos M. K. (2012). Identification, characterization and distribution of transposable elements in the flax (Linum usitatissimum l.) genome. BMC Genomics 13, 644. doi: 10.1186/1471-2164-13-644 23171245 PMC3544724

[B27] Grandbastien M.-A. (2015). LTR Retrotransposons, handy hitchhikers of plant regulation and stress response. Biochim. Biophys. Acta (BBA) - Gene Regul. Mech. 1849, 403–416. doi: 10.1016/j.bbagrm.2014.07.017 25086340

[B28] Havecker E. R. Gao X. Voytas D. F. (2004). The diversity of ltr retrotransposons. Genome Biol. 5, 1–6. doi: 10.1186/gb-2004-5-6-225 PMC463057 15186483

[B29] Ibrahim M. A. Al-Shomrani B. M. Alharbi S. N. Elliott T. A. Alsuabeyl M. S. Alqahtani F. H.. (2021). Genome-wide comparative analysis of transposable elements in palmae genomes. Front. bioscience (Landmark edition) 26, 1119–1131. doi: 10.52586/5014 34856758

[B30] Ito H. Yoshida T. Tsukahara S. Kawabe A. (2013). Evolution of the onsen retrotransposon family activated upon heat stress in brassicaceae. Gene 518, 256–261. doi: 10.1016/j.gene.2013 23370337

[B31] Jedlicka P. Lexa M. Kejnovsky E. (2020). What can long terminal repeats tell us about the age of LTR retrotransposons, gene conversion and ectopic recombination? Front. Plant Sci. 11. doi: 10.3389/fpls.2020.00644 PMC7251063 32508870

[B32] Jiang N. Bao Z. Zhang X. Eddy S. R. Wessler S. R. (2004). Pack-MULE transposable elements mediate gene evolution in plants. Nature 431(7008), 569–573. doi: 10.1038/nature02953 15457261

[B33] Jiao Y. Peluso P. Shi J. Liang T. Stitzer M. C. Wang B.. (2017). Improved maize reference genome with single-molecule technologies. Nature 546, 524–527. doi: 10.1038/nature22971 28605751 PMC7052699

[B34] Kalendar R. Vicient C. M. Peleg O. Anamthawat-Jonsson K. Bolshoy A. Schulman A. H. (2004). Large Retrotransposon derivatives: abundant, conserved but nonautonomous retroelements of barley and related genomes. Genetics 166, 1437–1450. doi: 10.1534/genetics.166.3.1437 15082561 PMC1470764

[B35] Kashkush K. Feldman M. Levy A. A. (2003). Transcriptional activation of retrotransposons alters the expression of adjacent genes in wheat. Nat. Genet. 33, 102–106. doi: 10.1038/ng1063 12483211

[B36] Kelly L. J. Renny-Byfield S. Pellicer J. Macas J. Novák P. Neumann P.. (2015). Analysis of the giant genomes of fritillaria (liliaceae) indicates that a lack of dna removal characterizes extreme expansions in genome size. New Phytol. 208, 596–607. doi: 10.1111/nph.13471 26061193 PMC4744688

[B37] Kijima T. E. Innan H. (2009). On the estimation of the insertion time of LTR retrotransposable elements. Mol. Biol. Evol. 27, 896–904. doi: 10.1093/molbev/msp295 19955475

[B38] Kimura M. (1980). A simple method for estimating evolutionary rates of base substitutions through comparative studies of nucleotide sequences. J. Mol. Evol. 16, 111–120. doi: 10.1007/BF01731581 7463489

[B39] Koch M. A. Haubold B. Mitchell-Olds T. (2000). Comparative evolutionary analysis of chalcone synthase and alcohol dehydrogenase loci in arabidopsis, arabis, and related genera (Brassicaceae). Mol. Biol. Evol. 17, 1483–1498. doi: 10.1093/oxfordjournals.molbev.a026248 11018155

[B40] Kumar A. Bennetzen J. L. (1999). Plant retrotransposons. Annu. Rev. Genet. 33, 479–532. doi: 10.1146/annurev.genet.33.1.479 10690416

[B41] Li S.-F. She H.-B. Yang L.-L. Lan L.-N. Zhang X.-Y. Wang L.-Y.. (2022). Impact of ltr retrotransposons on genome structure, evolution, and function in curcurbitaceae species. Int. J. Mol. Sci. 23, 10158. doi: 10.3390/ijms231710158 36077556 PMC9456015

[B42] Li S.-F. Zhang G.-J. Zhang X.-J. Yuan J.-H. Deng C.-L. Gu L.-F.. (2016). DPTEdb, an integrative database of transposable elements in dioecious plants. Database 2016, baw078. doi: 10.1093/database/baw078 27173524 PMC4865326

[B43] Liu Y. Yang G. (2014). Tc 1-like transposable elements in plant genomes. Mobile DNA 5, 17. doi: 10.1186/1759-8753-5-17 24926322 PMC4054914

[B44] Lockton S. Gaut B. S. (2009). The contribution of transposable elements to expressed coding sequence in arabidopsis thaliana. J. Mol. Evol. 68, 80–89. doi: 10.1007/s00239-008-9190-5 19125217

[B45] Luo X. Chen S. Zhang Y. (2022). Plantrep: a database of plant repetitive elements. Plant Cell Rep. 41, 1163–1166. doi: 10.1007/s00299-021-02817-y 34977976 PMC9035001

[B46] Ma J. Bennetzen J. L. (2004). Rapid recent growth and divergence of rice nuclear genomes. Proc. Natl. Acad. Sci. 101, 12404–12410. doi: 10.1073/pnas.0403715101 15240870 PMC515075

[B47] Ma B. Kuang L. Xin Y. He N. (2019). New insights into long terminal repeat retrotransposons in mulberry species. Genes 10, 285. doi: 10.3390/genes10040285 30970574 PMC6523491

[B48] Ma B. Li T. Xiang Z. He N. (2015). MnTEdb, a collective resource for mulberry transposable elements. Database 10, 1194 . doi: 10.1093/database/bav004 PMC4343074 25725060

[B49] Mann L. Seibt K. M. Weber B. Heitkam T. (2022). ECCsplorer: a pipeline to detect extrachromosomal circular DNA (eccDNA) from next-generation sequencing data. BMC Bioinf. 23, 40. doi: 10.1186/s12859-021-04545-2 PMC8760651 35030991

[B50] Marcon H. S. Domingues D. S. Silva J. C. Borges R. J. Matioli F. F. de Mattos Fontes M. R.. (2015). Transcriptionally active LTR retrotransposons in eucalyptus genus are differentially expressed and insertionally polymorphic. BMC Plant Biol. 15, 1–16. doi: 10.1186/s12870-015-0550-1 26268941 PMC4535378

[B51] Mokhtar M. M. Alsamman A. M. Abd-Elhalim H. M. El Allali A. (2021). CicerSpTEdb: A web-based database for high-resolution genome-wide identification of transposable elements in cicer species. PLos One 16, 1–21. doi: 10.1371/journal.pone.0259540 PMC8584679 34762703

[B52] Mokhtar M. M. Atia M. A. M. (2018). SSRome: an integrated database and pipelines for exploring microsatellites in all organisms. Nucleic Acids Res. 47, D244–D252. doi: 10.1093/nar/gky998 PMC6323889 30365025

[B53] Mokhtar M. M. EL Allali A. (2022). Pltrnadb: Plant transfer rna database. PLos One 17, 1–12. doi: 10.1371/journal.pone.0268904 PMC9126412 35605006

[B54] Neumann P. Novak P. Hoštáková N. Macas J. (2019). Systematic survey of plant LTR- retrotransposons elucidates phylogenetic relationships of their polyprotein domains and provides a reference for element classification. Mobile DNA 10, 1. doi: 10.1186/s13100-018-0144-1 30622655 PMC6317226

[B55] Orozco-Arias S. Jaimes P. A. Candamil M. S. Jiménez-Varón C. F. Tabares-Soto R. Isaza G.. (2021). Inpactordb: a classified lineage-level plant ltr retrotransposon reference library for free-alignment methods based on machine learning. Genes 12, 190. doi: 10.3390/genes12020190 33525408 PMC7910972

[B56] Ou S. Chen J. Jiang N. (2018). Assessing genome assembly quality using the ltr assembly index (lai). Nucleic Acids Res. 46, e126–e126. doi: 10.1093/nar/gky730 30107434 PMC6265445

[B57] Ou S. Jiang N. (2017). LTR Retriever: A highly accurate and sensitive program for identification of long terminal repeat retrotransposons. Plant Physiol. 176, 1410–1422. doi: 10.1104/pp.17.01310 29233850 PMC5813529

[B58] Ou S. Su W. Liao Y. Chougule K. Agda J. R. A. Hellinga A. J.. (2019). Benchmarking transposable element annotation methods for creation of a streamlined, comprehensive pipeline. Genome Biol. 20, 1–18. doi: 10.1186/s13059-019-1905-y 31843001 PMC6913007

[B59] Pedro D. L. F. Amorim T. S. Varani A. Guyot R. Domingues D. S. Paschoal A. R. (2021). An atlas of plant transposable elements. F1000Research 10. doi: 10.12688/f1000research.74524.1 PMC8729191 35035898

[B60] Pedro D. L. F. Lorenzetti A. P. R. Domingues D. S. Paschoal A. R. (2018). PlaNC-TE: a comprehensive knowledgebase of non-coding RNAs and transposable elements in plants. Database 2018, bay078. doi: 10.1093/database/bay078 30101318 PMC6146122

[B61] Pereira V. (2008). Automated paleontology of repetitive DNA with REANNOTATE. BMC Genomics 9, 614. doi: 10.1186/1471-2164-9-614 19094224 PMC2672092

[B62] Qu D. Sun W.-W. Li L. Ma L. Sun L. Jin X.. (2019). Long noncoding RNA MALAT1 releases epigenetic silencing of HIV-1 replication by displacing the polycomb repressive complex 2 from binding to the LTR promoter. Nucleic Acids Res. 47, 3013–3027. doi: 10.1093/nar/gkz117 30788509 PMC6451131

[B63] Rossi M. Araujo P. G. Van Sluys M.-A. (2001). Survey of transposable elements in sugarcane expressed sequence tags (ESTs). Genet. Mol. Biol. 24, 147–154. doi: 10.1590/S1415-47572001000100020

[B64] Sabot F. Sourdille P. Chantret N. Bernard M. (2006). Morgane, a new ltr retrotransposon group, and its subfamilies in wheats. Genetica 128, 439–447. doi: 10.1007/s10709-006-7725-5 17028971

[B65] Sato S. Tabata S. Hirakawa H. Asamizu E. Shirasawa K. Isobe S.. (2012). The tomato genome sequence provides insights into fleshy fruit evolution. Nature 485, 635. doi: 10.1038/nature11119 22660326 PMC3378239

[B66] Schnable P. S. Ware D. Fulton R. S. Stein J. C. Wei F. Pasternak S.. (2009). The B73 maize genome: complexity, diversity, and dynamics. science 326, 1112–1115. doi: 10.1126/science.1178534 19965430

[B67] Steinbiss S. Willhoeft U. Gremme G. Kurtz S. (2009). Fine-grained annotation and classification of *de novo* predicted ltr retrotransposons. Nucleic Acids Res. 37, 7002–7013. doi: 10.1093/nar/gkp759 19786494 PMC2790888

[B68] Szklarczyk D. Franceschini A. Wyder S. Forslund K. Heller D. Huerta-Cepas J. (2015). STRING v10: protein–protein interaction networks, integrated over the tree of life. Nucleic Acids Research 43 (D1), D447–D452. doi: 10.1093/nar/gku1003 25352553 PMC4383874

[B69] Tajima F. Nei M. (1984). Estimation of evolutionary distance between nucleotide sequences. Mol. Biol. Evol. 1 (3), 269–285. doi: 10.1093/oxfordjournals.molbev.a040317 6599968

[B70] Tanskanen J. A. Sabot F. Vicient C. Schulman A. H. (2007). Life without gag: The bare-2 retrotransposon as a parasite’s parasite. Gene 390, 166–117. doi: 10.1016/j.gene.2006.09.009 17107763

[B71] Thompson J. D. Gibson T. J. Higgins D. G. (2003). Multiple sequence alignment using clustalw and clustalx. Curr. Protoc. Bioinf. 00, 2.3.1–2.3.22. doi: 10.1002/0471250953.bi0203s00 18792934

[B72] Vitte C. Fustier M.-A. Alix K. Tenaillon M. I. (2014). The bright side of transposons in crop evolution. Briefings Funct. Genomics 13, 276–295. doi: 10.1093/bfgp/elu002 24681749

[B73] Wang H. Liu J.-S. (2008). LTR Retrotransposon landscape in medicago truncatula: more rapid removal than in rice. BMC Genomics 9, 382. doi: 10.1186/1471-2164-9-382 18691433 PMC2533021

[B74] Wang W. Zheng H. Fan C. Li J. Shi J. Cai Z.. (2006). High rate of chimeric gene origination by retroposition in plant genomes. Plant Cell 18, 1791–1802. doi: 10.1105/tpc.106.041905 16829590 PMC1533979

[B75] Wicker T. Gundlach H. Spannagl M. Uauy C. Borrill P. Ramírez-Gonzalez R. H.. (2018). Impact of transposable elements on genome structure and evolution in bread wheat. Genome Biol. 19, 103. doi: 10.1186/s13059-018-1479 30115100 PMC6097303

[B76] Wicker T. Matthews D. E. Keller B. (2002). TREP: a database for triticeae repetitive elements. Trends in plant science. 7(12), 561–2. doi: 10.1016/S1360-1385(02)02372-5

[B77] Wicker T. Sabot F. Hua-Van A. Bennetzen J. L. Capy P. Chalhoub B.. (2007). A unified classification system for eukaryotic transposable elements. Nat. Rev. Genet. 8, 973–982. doi: 10.1038/nrg2165 17984973

[B78] Witte C. P. Le Q. H. Bureau T. Kumar A. (2001). Terminal-repeat retrotransposons in miniature (TRIM) are involved in restructuring plant genomes. Proceedings of the National Academy of Sciences. 98(24), 13778–83. doi: 10.1073/pnas.241341898 PMC61118 11717436

[B79] Xu Z. Liu J. Ni W. Peng Z. Guo Y. Ye W.. (2017). GrTEdb: the first web-based database of transposable elements in cotton (Gossypium raimondii). Database 2017, bax013. doi: 10.1093/database/bax013.Bax013 28365739 PMC5467567

[B80] Xu Z. Wang H. (2007). LTR FINDER: an efficient tool for the prediction of full-length LTR retrotransposons. Nucleic Acids Res. 35, W265–W268. doi: 10.1093/nar/gkm286 17485477 PMC1933203

[B81] Yamamoto G. Miyabe I. Tanaka K. Kakuta M. Watanabe M. Kawakami S.. (2021). SVA retrotransposon insertion in exon of MMR genes results in aberrant RNA splicing and causes lynch syndrome. Eur. J. Hum. Genet. 29, 680–686. doi: 10.1038/s41431-020-00779-5 33293698 PMC8115629

[B82] Yi F. Jia Z. Xiao Y. Ma W. Wang J. (2018a). Sptedb: a database for transposable elements in salicaceous plants. Database 2018, bay024. doi: 10.1093/database/bay024 29688371 PMC5846285

[B83] Yi F. Ling J. Xiao Y. Zhang H. Ouyang F. Wang J. (2018b). ConTEdb: a comprehensive database of transposable elements in conifers. Database 2018, bay131. doi: 10.1093/database/bay131 30576494 PMC6301336

[B84] Zhang R.-G. Li G.-Y. Wang X.-L. Dainat J. Wang Z.-X. Ou S.. (2022). Tesorter: an accurate and fast method to classify ltr-retrotransposons in plant genomes. Horticulture Res. 9, uhac017. doi: 10.1093/hr/uhac017 PMC9002660 35184178

[B85] Zhao D. Ferguson A. A. Jiang N. (2016). What makes up plant genomes: The vanishing line between transposable elements and genes. Biochim. Biophys. Acta (BBA)-Gene Regul. Mech. 1859, 366–380. doi: 10.1016/j.bbagrm.2015.12.005 26709091

[B86] Zhou M. Hu B. Zhu Y. (2017). Genome-wide characterization and evolution analysis of long terminal repeat retroelements in moso bamboo (phyllostachys edulis). Tree Genet. Genomes 13, 1–12. doi: 10.1007/s11295-017-1114-3

[B87] Zhou S.-S. Yan X.-M. Zhang K.-F. Liu H. Xu J. Nie S.. (2021). A comprehensive annotation dataset of intact LTR retrotransposons of 300 plant genomes. Sci. Data 8, 174. doi: 10.1038/s41597-021-00968-x 34267227 PMC8282616

