# Supplementary figures and images for: PlantLTRdb: An interactive database for 195 plant species LTR-retrotransposons
Source: Front Plant Sci. 2023 Mar 6;14:1134627. doi: 10.3389/fpls.2023.1134627 (PMC10025401; doi:10.3389/fpls.2023.1134627)

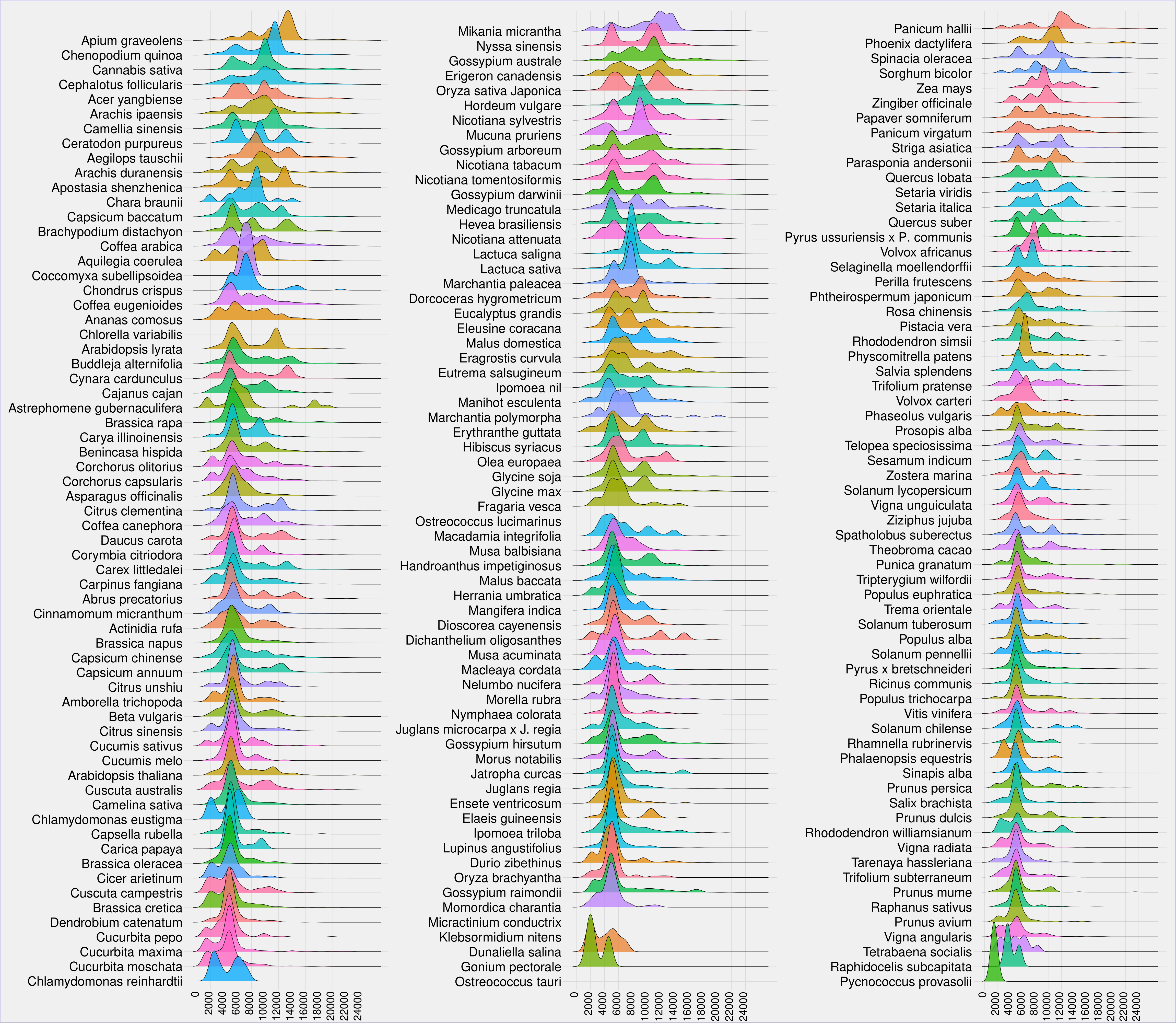

Supplement: Supplementary file 1 [file Image_1.png]

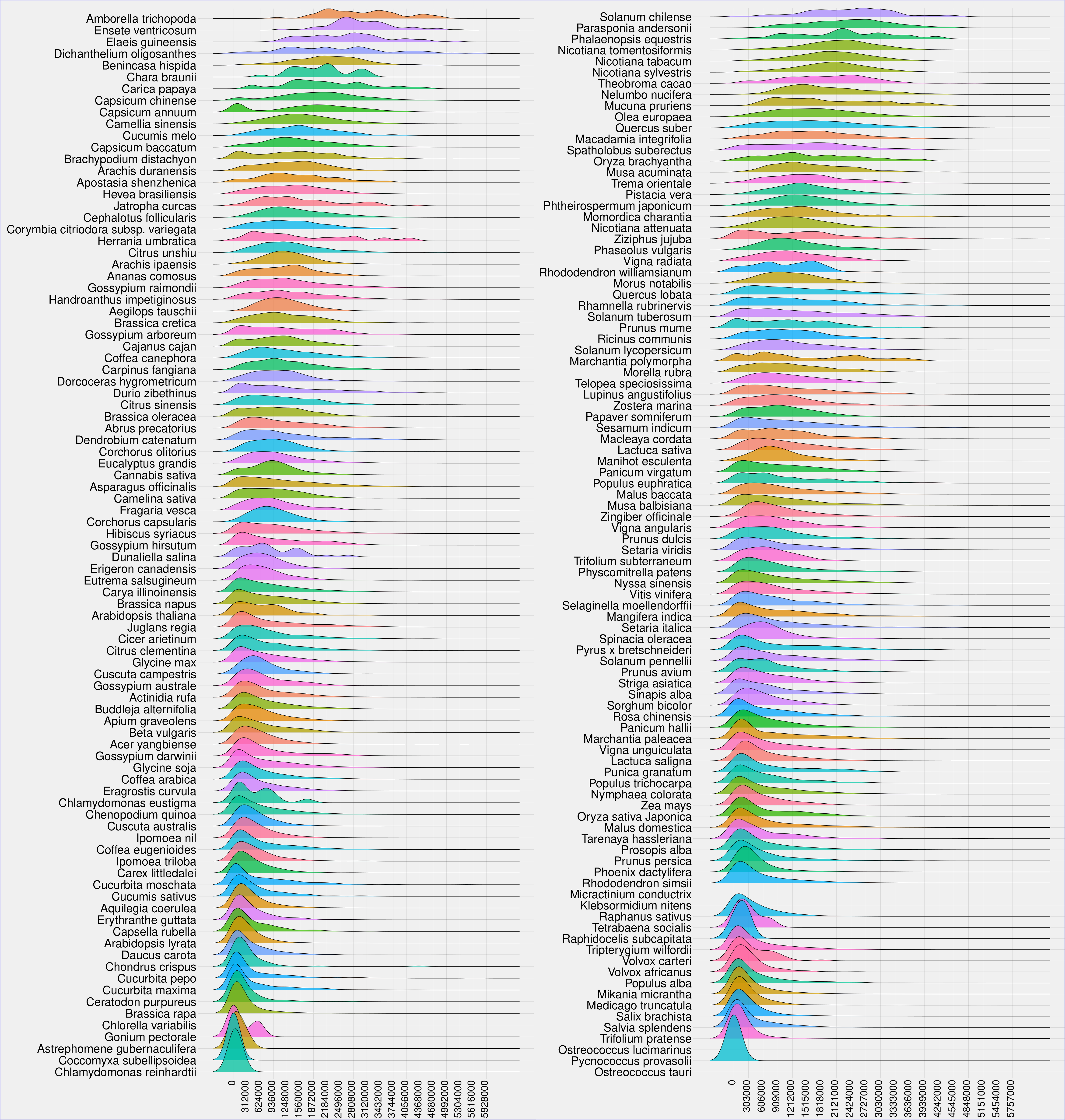

Supplement: Supplementary file 2 [file Image_2.png]

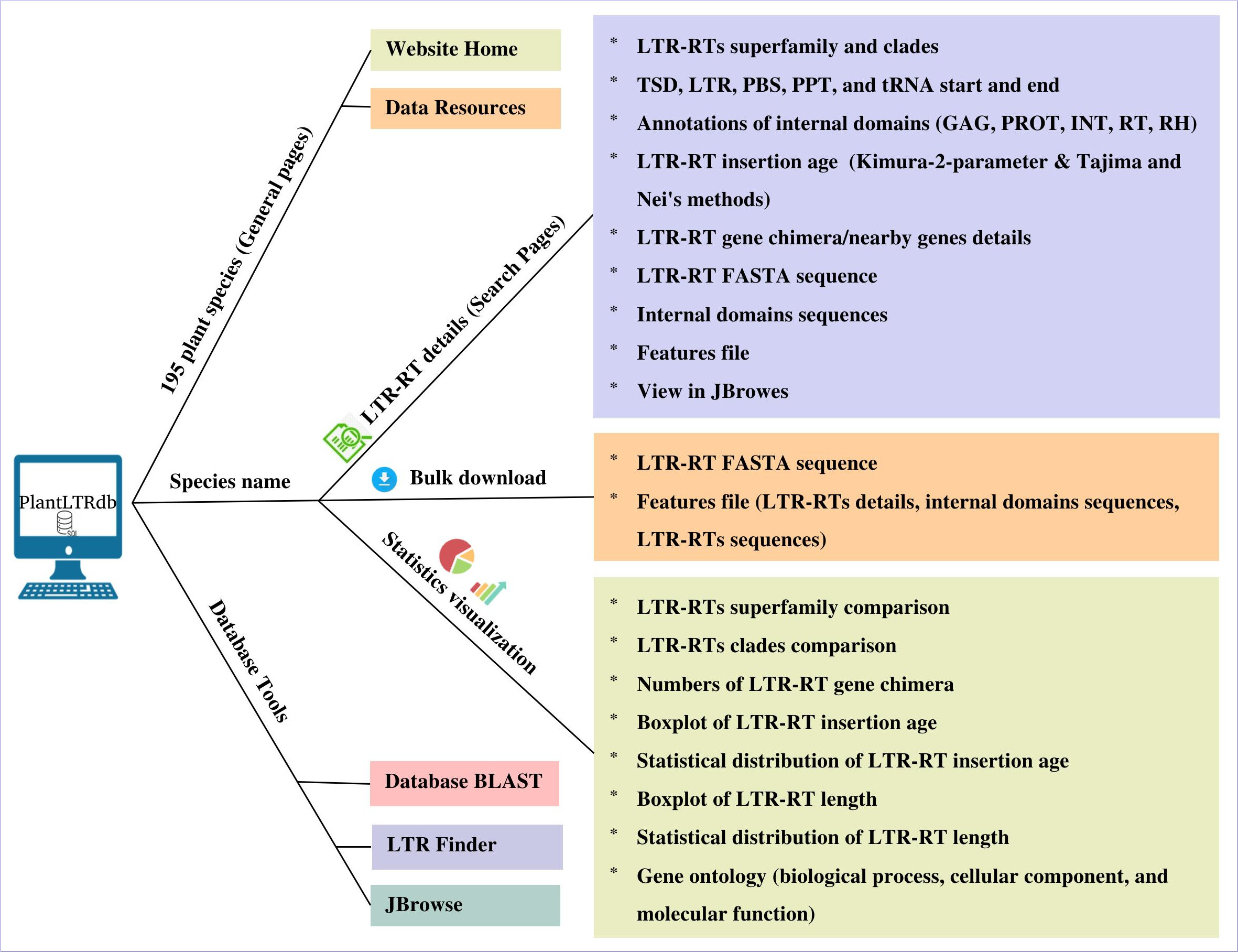

Supplement: Supplementary file 3 [file Image_3.jpeg]

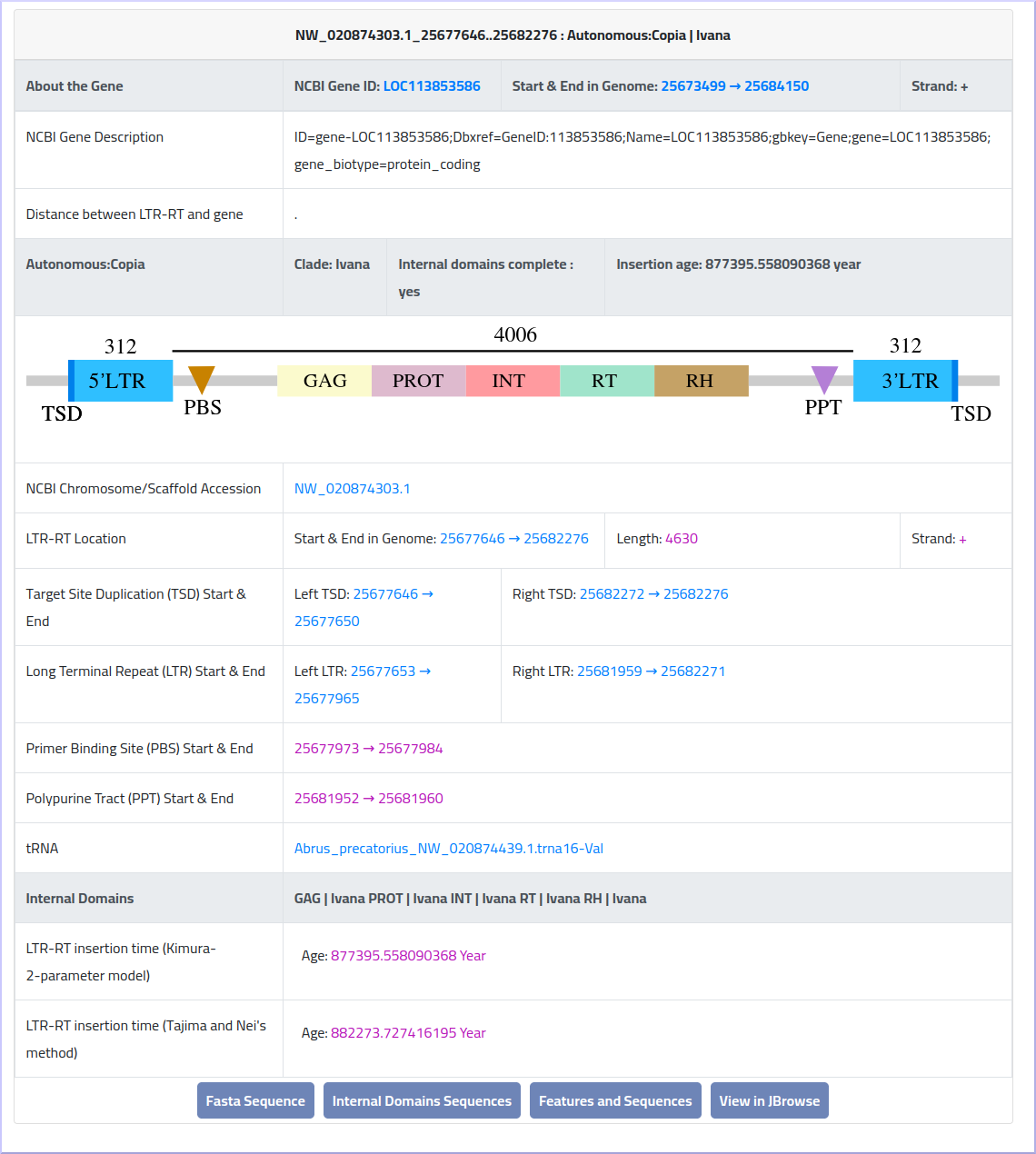

Supplement: Supplementary file 4 [file Image_4.png]
